# Structural and functional consequences in the amygdala of leptin-deficient mice

**DOI:** 10.1007/s00441-020-03266-x

**Published:** 2020-08-13

**Authors:** Jens Schepers, Christine Gebhardt, Alexander Bracke, Ina Eiffler, Oliver von Bohlen und Halbach

**Affiliations:** 1 Institut für Anatomie und Zellbiologie, Universitätsmedizin Greifswald, Friedrich Loeffler Str. 23c, 17487 Greifswald, Germany; 2 Institut für Neurophysiologie, Charité—Universitätsmedizin Berlin, Virchowweg 6, 10117 Berlin, Germany

**Keywords:** Amygdala, Dendritic spines, Leptin, LTP, Obesity

## Abstract

On the one hand, the emotional state can influence food intake and on the other hand, hunger can have an impact on the emotional state. Leptin, which is encoded by the ob gene, is involved in the energy homeostasis and plays a role in development of obesity. Mice deficient for leptin (ob/ob) are obese and display several behavioral alterations. It has been shown that ob/ob mice display striking changes in neuronal plasticity within the limbic system, e.g., hippocampal formation. We focus on alterations in ob/ob mice that can be related to alter processing in another part of the limbic system, the amygdala. ob/ob mice have a higher food consumption than age-matched controls, which might have an impact on the emotional state of these mice. Since the amygdala is involved in emotional processing, we analyze whether ob/ob mice display alterations in plasticity at the electrophysiological and structural level. No changes were seen in dendritic spine densities in the basolateral and lateral (LA) nucleus of the amygdala. Interestingly and in contrast to the hippocampus (Porter et al. 2013), long-term potentiation in the LA was increased in ob/ob mice. Our results indicate that amygdalar and hippocampal synaptic plasticity are regulated in different ways by leptin deficiency in accordance with the different functions of these limbic structures in stress and anxiety.

## Introduction

Obesity is associated with increased morbidity and mortality having a dramatic effect on individual and public health. Previous cross-sectional studies on body-weight-related alterations in brain structures revealed profound changes in white and gray matter resembling findings obtained from aged individuals (Mueller et al. 2015). Leptin is involved in the development of obesity and leptin deficiency (due to mutations in the ob gene) can be causal for obesity (Finger et al. 2010). Ob/ob mice, which were discovered in the 1950s (Ingalls et al. 1950), represent an animal model of obesity. At birth, ob/ob mice have nearly the same body weight as their controls but within 2 weeks postnatally, they become heavier. In contrast to diabetic leptin receptor (db/db)-deficient mice, blood glucose concentration is unaltered in ob/ob mice (Giesbertz et al. 2015). During the first 120 postnatal days, weight nearly doubles in ob/ob mice (Bracke et al. 2019). Moreover, they display deficits in hippocampal synaptic plasticity. An electrophysiological correlate of synaptic plasticity is represented by long-term potentiation (LTP). Hippocampal LTP is completely abolished in ob/ob mice (Porter et al. 2013), and adult hippocampal neurogenesis is also impaired (Bracke et al. 2019). Since LTP (Malenka and Nicoll 1999) and adult neurogenesis (Goncalves et al. 2016) have been linked to processes attributed to learning and memory, ob/ob mice may differ in hippocampus-related behavior. However, in contrast to db/db mice, which display deficits in the Morris water maze (Li et al. 2002), the ob/ob mice learn this task comparable with lean controls (Bracke et al. 2019). Despite the fact the leptin-deficient mice display normal appetitive spatial learning in the Y-maze (Finger et al. 2010), they developed increased immobility in the Porsolt test, an animal test model for depression (Collin et al. 2000). Moreover, ob/ob mice show anxiety-related behavior (Finger et al. 2010). Stress, depression and anxiety can lead to altered functioning of the hippocampus, the prefrontal cortex and the amygdala and vice versa (Leuner and Shors 2013; Mah et al. 2016; Qiao et al. 2014).

Following acute or chronic stress, LTP at the hippocampal synapses is impaired (Shors and Thompson 1992) or even abolished (Kumar 2011) and adult hippocampal neurogenesis is reduced (Dranovsky and Hen 2006). Rats subjected to chronic stress display enhanced LTP in the lateral nucleus (LA) of the amygdala (Suvrathan et al. 2014). The LA and the basolateral nucleus (BL) are members of the basolateral amygdala (De Olmos et al. 1985) and are involved in memory formation and emotional processing (Rolls 2000).

In this study, we analyze food consumption of ob/ob mice, since this may contribute to weight gain and behavioral and emotional alterations, which might be accompanied by changes in neuronal plasticity. The amygdala is involved in learning, memory and emotional processing. Moreover, evidence suggests that the amygdala is involved in the relevance processing of food stimuli and in cue-induced feeding (Coppin 2016). Thus, the emotional state has an impact on food intake and hunger can have an impact on the emotional state. We therefore analyze whether neuronal plasticity in the ob/ob mice is altered in the amygdala. Correlates of structural changes associated with neuronal plasticity, are, among others, changes in dendritic spine densities. An electrophysiological correlate of synaptic plasticity is LTP. Thus, ob/ob mice were analyzed with regard to changes in dendritic densities within the LA and BL. Moreover, since in ob/ob mice no LTP has been observed in the hippocampus, we tested whether LTP can be induced in the LA.

## Materials and methods

### Animals

Adult mice with the homozygous obese (ob/ob) spontaneous mutation (Ingalls et al. 1950) and their lean littermates were used (age between 4 and 6 month). B6.Cg-Lep^ob^/J mice were used, since in these mice hyperglycemia is only transient (Lindstrom 2007). Animals of both sexes were kept in a 12-h day-night cycle with food and water access ad libitum. The animal procedures were performed according to the guidelines for animal care and approved by the “Landesamtes für Landwirtschaft, Lebensmittelsicherheit und Fischerei Mecklenburg-Vorpommern (LALLF M-V)” and by the “Landesamt für Gesundheit und Soziales Berlin” (T0243/06).

### Food weight

Animals (control, *n* = 33; ob/ob = 30) were housed in cages with a defined portion of food. On the day before the food was renewed, the remaining food was weighted using a scale (ATILON, Precision Weighing Balances, USA). This weight was subtracted from the food weight in the beginning of the experiment. Thereafter, the mean food consumption per animal was calculated.

### Brain weight

Animals were euthanized and transcardially perfused with phosphate buffered saline (PBS) and thereafter with 4% paraformaldehyde (PFA). Thereafter, brains were removed and stored in PFA for 24 h. The next day, brains (*n* = 19 per group) were weighed using a scale (Beurer KS 36, Germany). To minimize errors, measurements were performed in triplicate and averaged.

### Analysis of dendritic spines

The brains were silver-impregnated using the Rapid GolgiStain reagent (FD NeuroTechnologies, USA) and 120-μm thick coronal sections were made. Analyses of the dendritic spines were conducted in a blinded fashion. Dendrites and their spines were reconstructed and analyzed using Neurolucida (Version 9.12, MBF Bioscience, USA) connected to an Axioplan 2 imaging microscope (Zeiss, Germany). 3D-reconstructions were done using a 100× oil objective (NA, 1.4; oil immersion), as described previously in detail (von Bohlen und Halbach et al. 2006). For each group, six brains were investigated. In each case, about 21 individual dendrites were mapped per region of the brain. The *n* values for the statistical analysis were based on animal numbers and not on numbers of analyzed elements.

### Slice preparation and electrophysiology

Mice (*n* = 4 per group) were anesthetized with 4% isoflurane and decapitated. Brains were quickly removed and transferred into ice-cold artificial cerebrospinal fluid (ACSF). Horizontal slices (400 μm) were made by using a vibroslicer (Campden Instruments, Silbey, UK). Slices were placed in an interface chamber, superfused continuously with ACSF and carbogenated with 95% O_2_ and 5% CO_2_. A bipolar stimulation electrode, placed into the external capsule (EC), was used for stimulation, as described previously (von Bohlen und Halbach and Albrecht 1998).

Recordings were made in different slices derived from one animal (*n*, number of slices). By varying single pulse stimulation intensities and averaging six responses per intensity, an input/output (I/O) response curve was constructed. The stimulus intensity that evoked a mean field potential equal to 50% of the maximum response was then used for subsequent stimulations (Zschenderlein et al. 2011). After I/O curve determination, single stimuli were applied for 30 min and evoked responses were monitored. Single stimuli (duration, 100 ms) were presented every 10 s. After obtaining stable baseline responses, we delivered high-frequency stimulation (HFS) as two trains at 100 Hz (duration, 1 s, 30 s apart), as described previously (Schubert and Albrecht 2008).

### Statistical analyses

Statistical analyses were performed using Prism 6.0 (GraphPad Software Inc., USA). Two-sided *t* test (food intake, brain weight) and non-parametric Mann Whitney test (dendritic spines) were used for statistical evaluation. Electrophysiological data were analyzed using two-way ANOVA (Gebhardt et al. 2019). Significance levels were set at *p* ≤ 0.05. Data were expressed as mean ± SEM.

## Results

### Food intake

**Fig. 1:**
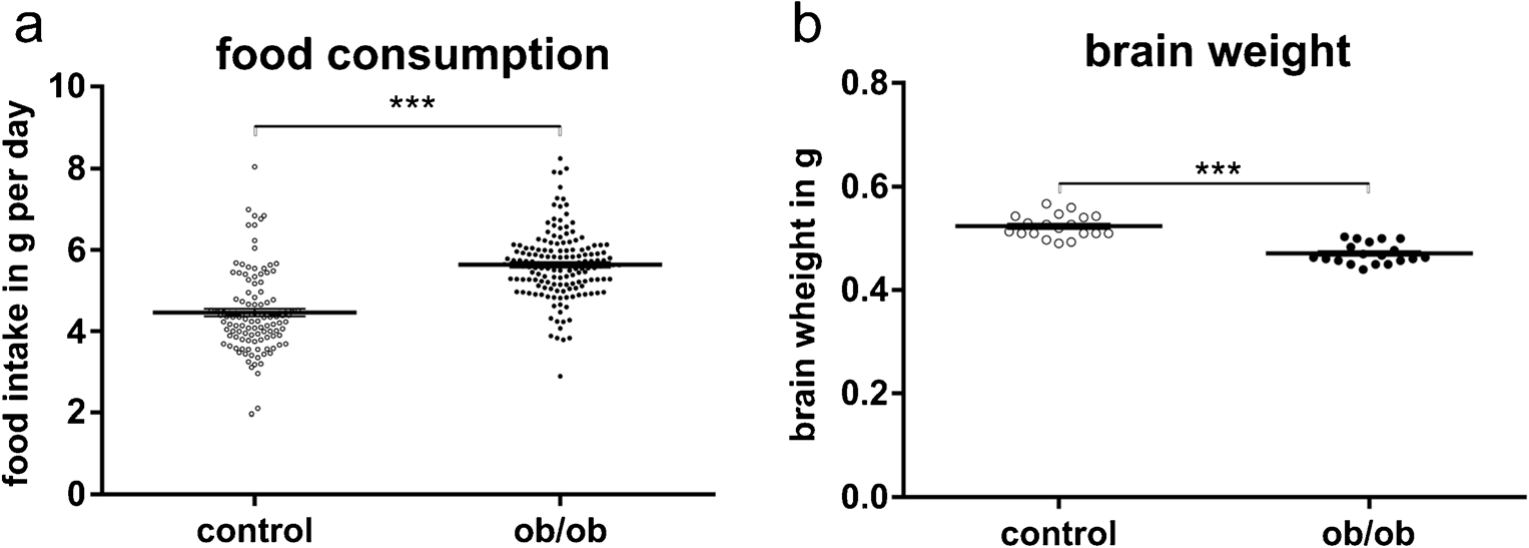
Food intake and brain weight. **a** Obese leptin-deficient mice (ob/ob; *n* = 30) display a higher food intake as controls (*n* = 33). **b** Brain weight of ob/ob mice (*n* = 19) is significantly lower as compared with controls (*n* = 19)

Leptin-deficient mice display a significant higher food consumption. Food intake was about 1.18 g per day higher than that of controls (ob/ob mice, 5.631 ± 0.814 g; control, 4.455 ± 0.985 g; *p* ≤ 0.001; age of animals, 127.8 ± 8 days; Fig. 1a).

### Brain weight

Despite of being obese, leptin-deficient mice display significantly reduced brain weight as compared with controls (ob/ob mice, 0.471 ± 0.02 g; control mice, 0.523 ± 0.022 g; *p* ≤ 0.001; age of animals, 129.2 ± 3 days; Fig. 1a).

### Dendritic spines

**Fig. 2:**
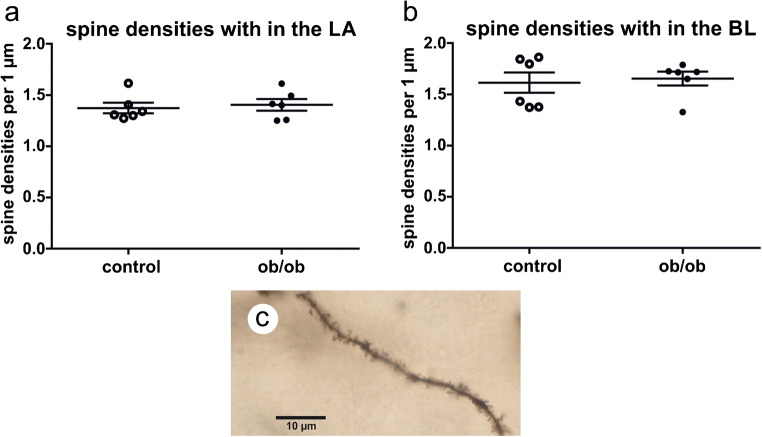
Analysis of dendritic spine densities within the LA and BL. **a** Dendritic spines’ densities within the LA did not show significant differences between the genotypes. **b** Densities of dendritic spines in the BL did not differ between controls and ob/ob mice. **c** A dendrite in the BL is covered with dendritic spines. Since the dendrite is not within a single focal plane, dendrites and dendritic spines were reconstructed and analyzed using Neurolucida

Dendritic spine densities were analyzed within the basolateral amygdala. Within the LA, spine densities have a tendency to be higher in ob/ob mice, (ob/ob mice, 1.405 ± 0.056; controls, 1.373 ± 0.052; *p* ≤ 0.898; Fig. 2a). Spine densities within the BL did not differ significantly (ob/ob mice, 1.654 ± 0.068; control mice, 1.614 ± 0.099; *p* ≤ 0.675; Fig. 2b, c).

### Long-term potentiation (LTP)

**Fig. 3:**
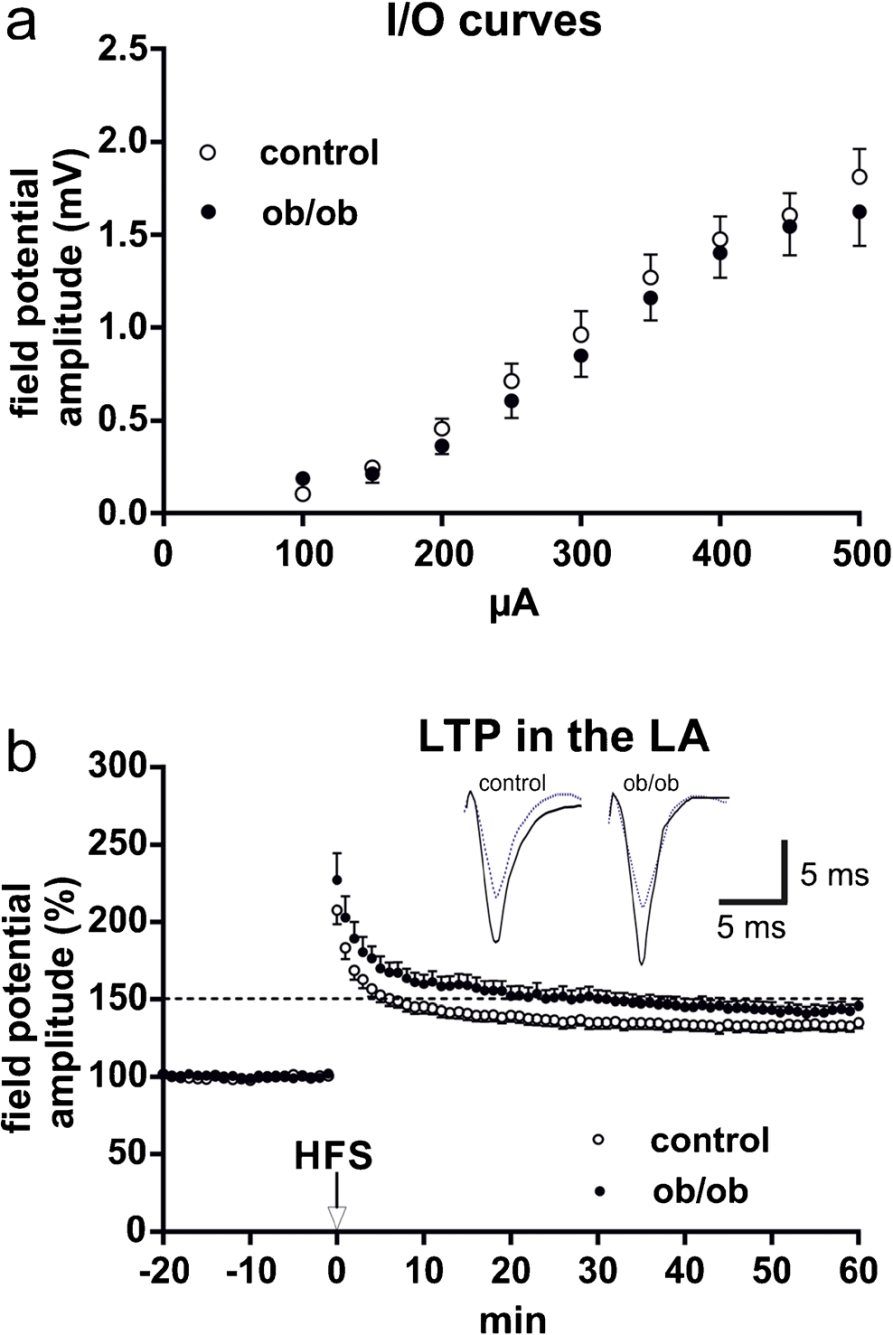
Baseline activity and LTP in the LA. **a** Input/output curves (I/O curves) of field potential amplitudes (evoked at EC fibers) recorded in the LA of ob/ob mice and controls (*n* = 18 slices per group). Basal transmission was not significantly different between the genotypes. **b** LTP can be induced by HFS of external capsule fibers in both, ob/ob mice and controls (*n* = 18 slices per group). In both cases, LTP was not only induced but also maintained over a longer time. The ob/ob mice display a stronger LTP than controls. Data points represent averaged amplitudes (mean ± SEM) normalized to baseline. Representative traces were recorded 5 min prior to tetanus (dashed lines) and 60 min after HFS (solid lines)

I/O curves were determined at the beginning of each experiment. No significant differences between the two genotypes were obvious (Fig. 3a). Robust LA-LTP could be induced in both groups by two tetanic stimuli applied at 100 Hz to fibers running through the EC. Thus, in contrast to the hippocampus, LTP could be induced in the ob/ob mice and this LTP was stable over time, comparable with the situation in controls. Interestingly, 1 h after LTP-induction, ob/ob mice display a nearly 10% higher field potential amplitude compared with controls (control, 132.1 ± 3.14 (*n* = 18); ob/ob, 144.2 ± 4.13 (*n* = 18); *p* = 0.0017; Fig. 3b).

## Discussion

Leptin-deficient mice show an obese phenotype that develops over time (Bracke et al. 2019) and this weight gain is associated with increased food intake. Despite their higher body weight, brain volume is reduced in ob/ob mice (Bracke et al. 2019). We also can confirm that brain weight is reduced in ob/ob mice (Ahima et al. 1999; Steppan and Swick 1999). In humans, an association between higher body mass index (BMI) and smaller brain volume has been reported (Carnell et al. 2012). Changes in brain size, especially reductions in hippocampal size, can be seen in major depression or after chronic stress in humans (Mervaala et al. 2000; Xia et al. 2004). Moreover, adult neurogenesis is reduced by stress, whereas chronic antidepressant treatment increases neurogenesis and blocks stress-induced effects (Dranovsky and Hen 2006). Reductions in adult hippocampal neurogenesis might, among others, contribute to brain volume reductions. In adult ob/ob mice, adult hippocampal neurogenesis is reduced (Bracke et al. 2019). The alteration in this parameter, together with others, might contribute to reductions in brain weight and size seen in the ob/ob mice.

In humans, there is an association of obesity with negative emotional states and brain areas like the hippocampus and amygdala that show high correlation between BMI and brain activity but only in the amygdala, the brain activity showed a correlation with negative emotional states (Park et al. 2017). LTP is completely abolished in the hippocampal CA1 region of ob/ob mice (Porter et al. 2013), whereas we could show that LTP is increased in the amygdala. These changes in synaptic plasticity might translate into morphological correlates of neuronal plasticity e.g., on the level of dendritic spines. However, spines’ densities within the hippocampal CA1 area were normal in ob/ob mice (Bracke et al. 2019).

In addition, no one-to-one relationship of dendritic spine densities and LTP in the amygdala is seen in the ob/ob mice. LTP influences not only spinogenesis but also spine morphology e.g., size of spine heads or postsynaptic densities (Bosch et al. 2014; Muller et al. 2000; Yuste and Bonhoeffer 2001). Furthermore, LTP can be influenced by changes in the level or spatial reorganization of postsynaptic proteins in spine heads (Rochefort and Konnerth 2012). Thus, it cannot be ruled out that, concerning dendritic spines, parameters, different from densities, were remodeled in the amygdala of ob/ob mice.

The amygdala is responsible for fear conditioning and the subsequent extinction. The amygdala also acts as a regulator of weight and intake behavior. Strong increases in food intake and body weight were observed in rats with lesions of the basolateral amygdala (Ganaraja and Jeganathan 2000). It is known that leptin receptors are expressed in the amygdala (Burguera et al. 2000; Udagawa et al. 2000), indicating that leptin may take part in the control not only of body weight but also of mood and emotion. Indeed, leptin has antidepressant-like and anxiolytic-like properties (Liu et al. 2010, 2017). Thus, leptin-deficiency results in marked alterations in anxiety-related behaviors (Finger et al. 2010). Anxiety is expressed as a long-lasting increase in synaptic strength in the LA and both, fear conditioning-induced neuronal plasticity and LTP at the amygdala synapses, seem to share common mechanisms of induction and expression (Bosch et al. 2014; Schroeder and Shinnick-Gallagher 2004). Our observation that LA-LTP is increased in ob/ob mice correlates with data describing a de-potentiation of LA-LTP in leptin-treated slices (Wang et al. 2015). Wang et al. (2015) also showed that leptin is involved in dampening the fear conditioning-induced synaptic potentiation in the LA through modulation of NMDA receptors. Our results also demonstrate that synaptic plasticity in the amygdala and in the hippocampus is regulated in different ways by leptin deficiency in accordance with the different functions of these limbic structures in stress and anxiety.
